# Enhancing Healthcare for People with Disabilities Through Artificial Intelligence: Evidence from Saudi Arabia

**DOI:** 10.3390/healthcare13131616

**Published:** 2025-07-06

**Authors:** Adel Saber Alanazi, Abdullah Salah Alanazi, Houcine Benlaria

**Affiliations:** 1 College of Education, Jouf University, Sakakah 72388, Saudi Arabia; asalanazi@ju.edu.sa; 2 King Salman Center for Disability Research, Riyadh 11614, Saudi Arabia; asdalananzi@ju.edu.sa; 3 Department of Clinical Pharmacy, College of Pharmacy, Jouf University, Sakaka 72341, Saudi Arabia; 4 College of Business, Jouf University, Sakakah 72388, Saudi Arabia

**Keywords:** artificial intelligence, healthcare accessibility, disability, Saudi Arabia, assistive technologies, Vision 2030, digital health

## Abstract

Background/Objectives: Artificial intelligence (AI) offers opportunities to enhance healthcare accessibility for people with disabilities (PwDs). However, their application in Saudi Arabia remains limited. This study explores PwDs’ experiences with AI technologies within the Kingdom’s Vision 2030 digital health framework to inform inclusive healthcare innovation strategies. Methods: Semi-structured interviews were conducted with nine PwDs across Riyadh, Al-Jouf, and the Northern Border region between January and February 2025. Participants used various AI-enabled technologies, including smart home assistants, mobile health applications, communication aids, and automated scheduling systems. Thematic analysis following Braun and Clarke’s six-phase framework was employed to identify key themes and patterns. Results: Four major themes emerged: (1) accessibility and usability challenges, including voice recognition difficulties and interface barriers; (2) personalization and autonomy through AI-assisted daily living tasks and medication management; (3) technological barriers such as connectivity issues and maintenance gaps; and (4) psychological acceptance influenced by family support and cultural integration. Participants noted infrastructure gaps in rural areas, financial constraints, limited disability-specific design, and digital literacy barriers while expressing optimism regarding AI’s potential to enhance independence and health outcomes. Conclusions: Realizing the benefits of AI for disability healthcare in Saudi Arabia requires culturally adapted designs, improved infrastructure investment in rural regions, inclusive policymaking, and targeted digital literacy programs. These findings support inclusive healthcare innovation aligned with Saudi Vision 2030 goals and provide evidence-based recommendations for implementing AI healthcare technologies for PwDs in similar cultural contexts.

## 1. Introduction

Artificial intelligence (AI) is reshaping healthcare delivery globally by offering unprecedented opportunities to improve diagnosis, rehabilitation, and patient management [1,2]. For people with disabilities (PwDs), AI presents potential solutions to long-standing barriers to accessing healthcare [3,4]. Despite international advancements, the application of AI in disability-related healthcare in Saudi Arabia remains underexplored.

The World Health Organization (WHO) estimates that over one billion people globally live with some form of disability, representing 15% of the world’s population [5]. In Saudi Arabia, demographic changes and increasing rates of chronic conditions have heightened the demand for assistive healthcare solutions [6,7]. AI-enabled technologies, including smart wheelchairs with obstacle recognition, mind-controlled exoskeletons, wearable health monitoring systems, brain–computer interfaces, and AI-enabled communication aids, could address these needs if implemented with cultural sensitivity [8,9].

The COVID-19 pandemic has highlighted the importance of digital healthcare systems for vulnerable populations, including PwDs [10,11]. Pandemic-related challenges for PwDs include deterioration of health conditions, exposure to lifestyle-related diseases, and restricted healthcare access [12,13]. Long-term isolation due to infection prevention measures creates additional barriers to accessing healthcare [14].

International studies have demonstrated AI’s potential for PwDs, from improving communication for the hearing-impaired to enhancing mobility for those with physical limitations [15,16,17]. However, ethical deployment, data privacy, affordability, and system interoperability issues often hinder adoption, particularly in culturally specific settings [18,19].

While AI adoption in healthcare is growing globally, Saudi Arabia faces unique challenges in implementing these technologies for people with disabilities (PwDs). Cultural values emphasizing family-centered healthcare decisions, Arabic language requirements for accessibility, and varying levels of digital infrastructure across regions create context-specific barriers that differ from implementation challenges documented in Western settings. Additionally, the kingdom’s rapid digital transformation under Vision 2030 has created opportunities alongside implementation gaps that require examination within Saudi cultural and geographic contexts.

Saudi Arabia has launched several digital health platforms as part of Vision 2030, including the Sehhaty, Mawid appointment, Wasfaty electronic prescription, and Sehha virtual consultation platforms [20,21]. While these initiatives represent important steps toward an integrated digital health ecosystem, their accessibility to PwDs remains questionable.

In healthcare settings, digital technology offers a potentially low-cost and scalable platform that can decrease the caregiver burden [22,23]. Caregivers of PwDs experience significant psychological and physiological stress [24], yet healthcare services for them remain insufficient [25,26]. As family support is a key element of health promotion among PwDs [27], comprehensive AI-enhanced systems should incorporate both PwDs and their caregivers.

Systems such as the iMHere (University of Pittsburgh) demonstrate the potential of three-way linkage systems that connect patients, caregivers, and physicians [16]. Similarly, the Electronic Patient-Reported Outcome (ePRO) System from the University of Toronto supports patients using comprehensive tracking tools [17].

Saudi Arabia’s unique cultural, infrastructural, and policy environment creates a distinct context for AI implementation. Urban–rural disparities [28], the central role of family in caregiving [6], cultural attitudes toward disability [29], and Vision 2030 transformation goals [20] influence how PwDs experience AI technologies.

Despite the rollout of national digital health platforms such as Sehhaty and Mawid, PwDs report accessibility gaps in these systems. Current platforms often lack screen reader compatibility, voice navigation options, and disability-specific user interface adaptations. The Sehhaty application, while offering appointment scheduling and health record access, presents navigation challenges for users with visual impairments and does not accommodate diverse disability-related healthcare needs. Similarly, telemedicine platforms introduced during COVID-19 lack features such as sign language interpretation or text-to-speech capabilities that would enhance accessibility for PwDs. These implementation gaps indicate that while digital health infrastructure exists, its design may not adequately address disability-specific requirements.

This research provides a qualitative understanding of how specific AI technologies are experienced by PwDs in Saudi Arabia. By capturing voices directly from the community, this study offers grounded insights to inform policy, practice, and future innovations in AI-enhanced healthcare for PwDs, contributing to research on inclusive healthcare innovation with context-specific recommendations.

## 2. Literature Review

### 2.1. AI Technologies in Healthcare for People with Disabilities

Artificial intelligence has demonstrated significant potential in addressing healthcare barriers for PwDs [15,30]. Various AI applications have emerged across disability types, including smart wheelchairs with obstacle recognition for individuals with physical disabilities, brain–computer interfaces for those with severe physical limitations, and AI-enabled communication aids for people with speech and hearing impairments [16,31].

For individuals with physical disabilities, AI-powered assistive technologies and enhanced wheelchair mobility systems have improved independence and safety. These technologies incorporate obstacle recognition, adaptive control, path planning, and navigation assistance, allowing users greater autonomy in healthcare settings [9,10]. Alanazi et al. [8] demonstrated that these technologies significantly reduce caregiver dependence when properly implemented.

Wearable health-monitoring devices show particular promise for individuals with physical and chronic conditions, enabling continuous health tracking, personalized rehabilitation protocols, and early intervention [22]. Although their effectiveness depends on reliable connectivity and proper integration with healthcare systems, these devices can transmit vital health information to healthcare providers, while accommodating mobility limitations [18].

Brain–computer interfaces (BCIs) and mind-controlled exoskeletons represent revolutionary advancements for individuals with severe physical disabilities. These technologies translate neural signals into digital commands, allowing the direct control of assistive devices through thought alone [32]. While promising, these advanced technologies raise unique ethical considerations regarding neural data privacy and psychological adaptation [33].

Smart home assistants support individuals with physical and cognitive disabilities by enabling voice-activated control of the environment and healthcare reminders. Research indicates that these systems improve medication adherence and appointment attendance when properly configured [34]. However, emergency healthcare command recognition remains a challenge, particularly in multilingual contexts [35].

Despite these advancements, technological barriers persist, including limited interoperability between healthcare systems, inadequate digital infrastructure in rural areas, and high implementation costs [10,28]. Ethical concerns, including data privacy, algorithmic bias, and informed consent issues, are particularly pronounced when working with vulnerable populations [3].

### 2.2. Disability Healthcare Context in Saudi Arabia

Saudi Arabia’s healthcare system is undergoing significant transformation under Vision 2030, which aims to improve healthcare quality, accessibility, and efficiency [20]. Recent estimates suggest that 7.1% of the Saudi population lives with some form of disability [6], yet they often face disproportionate barriers to accessing healthcare.

During the COVID-19 pandemic, these challenges were exacerbated because infection prevention measures created additional complications in those requiring mobility support [10]. The sudden discontinuation of in-person services caused significant disruption among PwDs who relied on this support [13].

Traditional healthcare barriers for PwDs in Saudi Arabia include limited physical accessibility of facilities, inadequate disability training among healthcare providers, and centralization of specialized services in urban areas [28]. These challenges are compounded by stigma and discrimination, which can deter individuals from seeking necessary care [29].

In response, Saudi Arabia initiated several programs aimed at enhancing healthcare accessibility for PwDs, including the National Transformation Program and the Human Capability Development Program [7]. However, Alasiri and Mohammed [36] indicate that these initiatives have not yet fully addressed the specific accessibility needs of PwDs, particularly concerning digital and AI-enhanced services.

### 2.3. Cultural and Contextual Barriers to AI Adoption in Saudi Arabia

Saudi Arabia’s healthcare digitalization has accelerated with significant investments in technological infrastructure [20]. Various platforms have been launched, including the Sehhaty application, Mawid appointment system, Wasfaty electronic prescription service, and Sehha virtual consultation platform [21]. The COVID-19 pandemic catalyzed digital healthcare adoption, with telehealth services experiencing a 318% increase in utilization by 2020 [36]. While these initiatives represent important steps toward an integrated digital health ecosystem, accessibility for disabled users varies considerably.

Despite this digital transformation progress, research examining AI healthcare adoption within Saudi contexts reveals cultural and systemic barriers that differ from international implementation experiences. Alanzi et al. [37] identified key obstacles to AI adoption among family medicine physicians in Saudi Arabia, including concerns about maintaining traditional patient–physician relationships and integration challenges with existing healthcare workflows. These findings highlight tensions between technological advancement and established medical practice patterns valued within Saudi healthcare culture.

Healthcare professionals’ acceptance of AI technologies is influenced by trust and confidence factors specific to local contexts. Althubaiti and Al Yousef [38] found that healthcare workers expressed concerns about AI system reliability and questioned whether these technologies could adequately understand Arabic medical terminology and cultural health concepts. Language barriers emerged as a significant obstacle, with existing AI systems often lacking appropriate Arabic language processing capabilities for medical contexts.

Professional cultural dynamics further complicate AI implementation. Ramadan et al. [39] reported that registered nurses identified organizational hierarchy concerns and unclear role definitions as obstacles to AI adoption, reflecting cultural workplace dynamics that may limit technology integration. These findings suggest that AI implementation requires consideration of professional cultural norms alongside technical capabilities.

Broader concerns about AI adoption among Saudi healthcare professionals include data security and patient privacy considerations that intersect with cultural values. Alsaedi et al. [40] documented healthcare workers’ apprehensions about patient data management and the potential for AI systems to conflict with Islamic principles regarding privacy and confidentiality. Additionally, participants expressed concerns about reduced human interaction in healthcare delivery, which contradicts cultural values emphasizing personal relationships in medical care.

Geographic disparities compound these cultural barriers. While urban centers have achieved relatively high digital healthcare adoption rates, rural areas continue to face significant barriers related to internet connectivity, digital literacy, and accessibility of support services [41]. Saudi Arabia has begun developing three-way linkage systems, similar to the iMHere system (University of Pittsburgh) and the ePRO system (University of Toronto), which connect patients, caregivers, and healthcare providers [16,17]. However, there remains no health management service that effectively records the health information of both PwDs and their caregivers [10].

The intersection of AI technologies and disability healthcare within Saudi Arabia’s digital transformation continues to emerge. Preliminary initiatives include AI-powered diagnostic tools, virtual rehabilitation platforms, and remote monitoring systems [9]. However, evaluations of the effectiveness, accessibility, and cultural appropriateness of these technologies for PwDs remain limited [42].

### 2.4. Theoretical Frameworks for AI Integration in Disability Healthcare

Several theoretical frameworks provide valuable perspectives for understanding AI integration in healthcare for disabled people.

The Technology Acceptance Model (TAM) highlights the importance of perceived usefulness and ease of use in technology adoption [43]. This framework is particularly relevant when considering how people with different disabilities might engage in AI-based healthcare solutions [1].

The Digital Inclusion Framework (DIF) emphasizes creating inclusive environments that address the specific challenges faced by marginalized groups [44]. This framework advocates participatory design approaches that directly involve PwDs in technology development [31].

The Health Accessibility Model (HAM) focuses on the structural, social, and personal factors that influence healthcare access, highlighting the importance of addressing multiple barriers simultaneously [45]. When applied to AI in healthcare, this model emphasizes holistic approaches that concurrently address technological, social, and structural barriers.

The integration of these frameworks, as suggested by Johansson et al. [32], can provide a comprehensive foundation for evaluating and improving AI healthcare solutions in complex cultural contexts, such as Saudi Arabia.

### 2.5. Support Systems and Healthcare Providers

In the Saudi context, the traditional healthcare system relies heavily on in-person interactions, with limited digital integration. Healthcare professionals’ attitudes toward and familiarity with digital technologies significantly influence adoption rates among disabled patients [46].

Family caregiver involvement increases patient satisfaction and adherence [26]. This is particularly relevant in Saudi Arabia, where family structures play a central role in healthcare decision making. Family caregivers of patients with chronic conditions experience significant psychological and physiological stress, highlighting the need for support systems that address caregivers’ needs alongside those of PwDs [24].

Professional coordination and structured support programs can help patients navigate healthcare systems while also building resilience. Community support services enhance caregivers’ role in improving patient care [25]. Various services using information and communication technology are being developed to support PwDs, although these services are often implemented without sufficient consideration of caregivers’ needs [10].

In their three-way digital healthcare system evaluation, Kim et al. [10] demonstrated that a system architecture addressing complexity, convenience, professionalism, learnability, and simplicity significantly improved usability across PwDs, caregivers, and healthcare professionals.

### 2.6. Barriers and Facilitators

Access to digital healthcare is influenced by technological, systemic, and cultural factors. Marginalized populations face preparedness and device access barriers [35], whereas healthcare professionals encounter systemic challenges when implementing new technologies [18]. These barriers are particularly pronounced in rural areas where both infrastructure limitations and cultural factors impede technology adoption.

Cultural factors include trust in technology, which correlates with willingness to use digital services [19]. Almulhem [46] identified specific cultural barriers to mobile health acceptance among the older Saudi population, including concerns about replacing human care with technology, privacy considerations within family structures, and religious perspectives on health management.

Community support and targeted training programs can help individuals overcome barriers to digital health technology adoption [47,48]. The effectiveness of these supports varies based on the local context, with rural communities often requiring more intensive interventions to achieve successful technological integration [49].

Government support serves as a crucial mediating factor in technology adoption among PwDs in Saudi Arabia. Policy guidelines and state-sponsored aid significantly influence assistive technology diffusion, suggesting that similar approaches may be effective for healthcare-related AI technologies [8].

#### 2.6.1. Primary Research Question

How do people with disabilities in Saudi Arabia experience AI technologies in healthcare and what factors influence their accessibility and effectiveness?

#### 2.6.2. Secondary Research Questions

1. What patterns of AI technology use have emerged across different disability types in the Saudi healthcare context?
2. How do geographic location and digital literacy influence access to AI-enhanced healthcare technologies?
3. What role do support systems play in facilitating effective use of AI healthcare technologies?
4. How do cultural factors specific to Saudi Arabia affect the acceptance of AI technologies in disability healthcare?
5. What barriers and facilitators exist for various AI technologies within the Saudi healthcare system?

#### 2.6.3. Research Problem Statement

Saudi Arabia’s Vision 2030 has promoted digital healthcare transformation through AI technology investments. However, limited research has examined how these developments affect people with disabilities (PwDs), creating a knowledge gap regarding AI healthcare access and adoption in this population.

The current literature on AI healthcare for PwDs exhibits several limitations. Most research focuses on Western populations [10,22], with limited examination of how cultural values and family structures influence technology adoption in non-Western contexts. Studies typically examine individual AI technologies rather than user experiences across multiple applications [43], revealing limited information about how PwDs navigate AI healthcare systems. Additionally, research emphasizes technical functionality over user acceptance factors, particularly cultural and psychological dimensions that affect sustained adoption [16].

The literature also lacks examination of geographic disparities within countries. While studies document urban–rural digital divides generally [28], research has not explored how these disparities affect AI healthcare adoption for PwDs across different regional contexts.

This study addresses these gaps by examining PwDs’ experiences with multiple AI healthcare technologies across Saudi regions, focusing on user acceptance factors and cultural considerations rather than technical performance. The findings inform implementation strategies that support equitable AI healthcare access for PwDs in Saudi Arabia and similar contexts.

## 3. Methods

### 3.1. Study Design

This study employed a qualitative phenomenological approach to explore how artificial intelligence technologies impact the healthcare experiences of people with disabilities (PwDs) in Saudi Arabia. This study was approved by the National Committee of Scientific Research (NCSR) at Jouf University (approval number: HAP-13-S-001). Semi-structured interviews were conducted between January and February 2025, featuring open-ended questions designed to elicit rich descriptions of personal experiences with AI healthcare technologies.

The sample size of nine participants was determined by thematic saturation principles, where data collection continued until no new themes emerged from subsequent interviews. Saturation was achieved after the seventh interview, with two additional interviews conducted for confirmation. While thematic saturation was reached within this sample, the study’s generalizability is limited by the focused geographic representation (three regions) and specific disability types included. Future studies should expand to broader demographics, including additional Saudi regions, diverse disability categories, and varying socioeconomic backgrounds to enhance transferability of findings across the kingdom’s diverse population contexts.

### 3.2. Data Collection Plan and Rationale

Semi-structured interviews provided in-depth qualitative data, allowing participants to articulate their experiences, perceptions, and challenges of using AI-enhanced healthcare technologies. This methodology is particularly suitable for exploring the complex interplay between disabilities, technology, cultural contexts, and healthcare systems. Interviews were conducted in Arabic or English according to participant preference, with each session lasting 45–60 min. The questions covered the awareness and usage of AI healthcare tools, perceived benefits and limitations, accessibility concerns, and ethical considerations.

#### Thematic Analysis Procedure

This study employed Braun and Clarke’s [50] six-phase thematic analysis framework. The three stages were conducted as follows:

1. Stage 1: Data Familiarization and Initial Coding (January 2025)—All interviews were transcribed verbatim in Arabic and translated to English. Two researchers independently read transcripts multiple times. Initial codes were generated inductively from participant narratives. One hundred twenty-seven preliminary codes were identified across nine transcripts.
2. Stage 2: Theme Development and Review (February 2025)—Related codes were clustered into potential themes. Candidate themes were reviewed against coded extracts and entire dataset. Four main themes emerged with 12 sub-themes. Inter-rater reliability achieved 89% agreement between coders.
3. Stage 3: Theme Refinement and Definition (February 2025)—Themes were refined through iterative discussion. Each theme was clearly defined with scope and boundaries. Connections to the existing literature were established. Representative quotes were selected for each theme.

### 3.3. Study Sample Selection

While this sample size aligns with qualitative research standards for phenomenological studies, the geographic and demographic scope may limit transferability to broader PwDs populations in Saudi Arabia.

Purposive sampling was used to recruit participants from Saudi Arabian disability associations and healthcare centers to ensure diverse disability types, geographic locations, and AI technology experiences. For geographic diversity in technology access, participants were drawn from Riyadh (the urban capital), Al-Jouf, and the Northern Border region, representing both urban and rural areas.

A sample size of nine participants proved sufficient for this qualitative research, with thematic saturation reached after the eighth interview when no new themes or insights emerged. This indicates that the relevant experiences, barriers, and perceptions of the research questions were adequately captured. Purposive selection ensures diversity in disability types, digital literacy levels, and geographic representation, thus enriching the findings.

The sample size aligns with established qualitative research practices for phenomenological studies, where data saturation typically occurs within 6–12 participants [51] (Guest et al., 2020). Interview analysis indicated that key themes emerged consistently across participants, suggesting adequate depth for initial theory development. However, the limited geographic and demographic diversity within the sample may have constrained the identification of region-specific or subgroup-specific adoption patterns.

### 3.4. Participant Characteristics

The final study included nine participants with disabilities. The study population consisted of individuals with severe physical disabilities, chronic conditions, neurological disabilities, cognitive disabilities, hearing impairments, and speech impairments. Participants had varying levels of digital literacy (high, moderate, or low).

Participation in the study was voluntary and was unpaid. All participants were briefed on the goals and methods of the study before the interviews. The researcher maintained confidential data and informed the participants that they could withdraw at any time without penalty. All participants provided informed consent through their signatures, with appropriate communication accommodation provided as needed.

Table 1 provides demographic information for all participants, including gender, age, region, type of disability, AI technology used, and digital literacy level.

#### Interview

The interviews were conducted in Arabic, where the respondents discussed artificial intelligence healthcare applications for people with disabilities in Saudi Arabia, digital healthcare access barriers, and support systems. Table 2 presents the interview questions, their purposes, and references to the relevant studies.

### 3.5. Researcher Positionality

The multidisciplinary research team represented education, clinical pharmacy, and business backgrounds, potentially influencing focus toward digital literacy, medical applications, and implementation considerations, respectively. The team’s regional institutional affiliation facilitated participant recruitment within Al-Jouf but may have influenced the interpretation of challenges in other study regions. As Arabic-speaking Saudi researchers, the team shared a cultural and linguistic context with participants, potentially enhancing openness while creating assumptions about cultural norms.

To address potential biases, interview transcripts were reviewed by multiple team members, participant validation was conducted, and reflexive discussions documented disciplinary assumptions throughout the analysis process.

## 4. Results

Thematic analysis of interviews with nine individuals with various disabilities across Saudi Arabia revealed four major themes related to AI in healthcare: (1) accessibility and usability of AI tools, (2) personalization and autonomy, (3) technological barriers, and (4) psychological acceptance. These themes reflect the complex relationship between AI technologies, healthcare systems, and lived experiences of people with disabilities (PwDs) in the Saudi context.

### 4.1. Accessibility and Usability of AI Tools

This theme encompasses the fundamental barriers that prevent PwDs from effectively accessing and utilizing AI healthcare technologies. It emerged from 34 coded segments across all nine interviews, reflecting three distinct but interconnected challenges: geographic access disparities, disability-specific design deficiencies, and assistive technology compatibility issues.

These findings directly engage with digital health equity frameworks [35,44] while extending this work into the specific context of AI-enhanced healthcare technologies. Our results contribute to ongoing debates about universal design principles in healthcare AI and challenge assumptions about technology accessibility in resource-diverse settings.

#### 4.1.1. Geographic Disparities in AI Healthcare Access

Geographic location emerged as a primary determinant of AI healthcare access, confirming and extending [10] findings on rural–urban digital divides in healthcare. However, our data reveals that accessibility challenges go beyond simple connectivity issues to encompass specialized technical support and maintenance infrastructure:

“My AI-enhanced wheelchair with autonomous navigation capabilities has transformed my independence, but the maintenance costs are substantial. When environmental sensors need recalibration or the path-planning software requires updates, I often have to delay owing to costs.”

(P4: physical disability, Al-Jouf)

This finding contributes to current debates in the AI-disability literature about sustainable implementation in diverse geographic contexts. While [28] identify general e-health infrastructure challenges in Saudi Arabia, our results demonstrate how AI healthcare technologies create additional layers of geographic inequality that extend beyond initial access to include ongoing technical support requirements.

#### 4.1.2. Disability-Specific Design Deficiencies:

Participants consistently reported that available AI applications lack disability-specific customization, directly challenging the “universal” applicability claims prevalent in healthcare AI marketing. This finding aligns with the critique in [1] of one-size-fits-all approaches while revealing additional complexity specific to Arabic language processing:

“The AI speech recognition system struggles with my speech patterns. Despite being marketed specifically for speech impairments, it requires constant retraining to understand my voice.”

(P9: speech impairment, Northern Border)

This quote illustrates a critical gap in current AI-disability research, which has focused primarily on English-language systems. Our findings extend the call in [15] for culturally adapted AI systems by demonstrating how linguistic and disability factors intersect to create compound accessibility barriers not addressed in existing universal design frameworks.

#### 4.1.3. Assistive Technology Compatibility

The incompatibility between AI healthcare tools and existing assistive technologies emerged as a significant barrier, reflecting broader interoperability issues documented in the healthcare AI literature [18]. However, our findings reveal how these technical challenges disproportionately affect PwDs who rely on multiple interconnected assistive systems:

“My AI-powered navigation assistance generates personalized routes, but the interface isn’t compatible with my screen reader, so I need assistance to interpret the directions.”

(P2: visual impairment, Riyadh)

This finding contributes to ongoing debates about healthcare AI integration by demonstrating how interoperability failures can actually increase dependency rather than enhance autonomy—a critical consideration for AI-disability applications that contradicts the empowerment rhetoric common in this field.

#### 4.1.4. Successful Integration Patterns: Design Features That Work

Despite widespread challenges, participants who successfully utilized AI healthcare tools identified specific design features that enhanced accessibility. These success factors provide concrete guidance for addressing the barriers identified above:

“My smart wheelchair with AI obstacle recognition has transformed my hospital visits. The integration with the hospital’s navigation system guided me directly to my appointments.”

(P3: physical disability, Riyadh)

Analysis of successful integration patterns reveals three critical design principles: (1) seamless integration with existing assistive technologies, (2) multiple communication modalities (voice, text, visual), and (3) cultural and linguistic adaptation. These findings extend the work in [16] on accessible AI design by providing empirical evidence from a non-Western context.

#### 4.1.5. Technology Distribution Patterns Across Disability Types

The distribution of AI technologies across disability types reveals specific patterns of application and utility that illuminate both opportunities and gaps in current AI-disability healthcare provision (Table 3).

The technology distribution shown in Table 3 reveals several important patterns that contribute to broader discussions about AI-disability healthcare equity:

1. Physical Disability Overrepresentation: physical disabilities (PD, SPD) account for 33% of participants but utilize the most sophisticated AI technologies (BCI, AI-EWM), suggesting that current AI development prioritizes mobility-related applications.
2. Sensory Disability Underservicing: despite well-documented needs, participants with sensory disabilities (VI, HI, SI) reported limited AI tool options, indicating a critical gap in development priorities.
3. Cognitive Disability Marginalization: only one participant with cognitive disabilities was included, and available technologies (SHA) were basic compared to those available for physical disabilities, reflecting broader patterns of exclusion in healthcare AI development.

These patterns contribute to current debates about healthcare AI equity by demonstrating how development priorities may inadvertently recreate existing healthcare disparities within PwDs communities.

### 4.2. Personalization and Autonomy

This theme examines the relationship between AI personalization capabilities and user autonomy in healthcare settings. It emerged from 28 coded segments across seven interviews, encompassing technology-mediated empowerment, personalization challenges, and decision-making authority.

These findings relate to the work in [16] on technology-mediated empowerment and extend [1] the Technology Acceptance Model into cross-cultural healthcare contexts. The results contribute to discussions about AI personalization in healthcare [22] while examining how concepts of individual autonomy may vary across cultural contexts.

#### 4.2.1. Technology-Mediated Empowerment

AI technologies demonstrated potential for enhancing user autonomy, particularly for participants with severe physical limitations:

“My brain-computer interface system has given me unprecedented independence. I can control my medical devices, communicate with healthcare providers, and access my health records based on my thoughts.”

(P5: severe physical disability, Riyadh)

This finding relates to the assistive technology empowerment literature [8] by showing how AI systems can restore functional capacity and decision-making authority. However, unlike studies in Western contexts that frame this as individual empowerment, the Saudi context reveals how technological independence intersects with family-centered healthcare approaches.

#### 4.2.2. Adaptive Personalization Challenges

Participants valued AI systems that adapted to their changing needs, though implementation varied:

“The AI-driven rehabilitation technology creates personalized exercise programs based on my progress. It adapts in real time to my capabilities, pushing me just enough without causing strain or injury.”

(P6: physical and neurological disabilities, Al-Jouf)

This example extends the framework in [22] for adaptive healthcare technologies. However, successful personalization appears to require cultural and contextual understanding beyond technical adaptation—a dimension less explored in the current AI-disability literature.

#### 4.2.3. Personalization Limitations

Despite personalization promises, participants encountered systems that failed to accommodate their requirements:

“My smart home assistant isn’t programmed to understand emergency health commands with sufficient nuance. It struggles to differentiate between requesting regular medication and needing emergency assistance.”

(P8: physical and cognitive disabilities, Northern Border)

This finding relates to debates about AI standardization versus customization [18] by showing how insufficient personalization can increase vulnerability. The inability to distinguish between routine and emergency requests represents a safety concern often overlooked in AI-disability research focused on daily living improvements.

#### 4.2.4. Human–AI Decision-Making Balance

Participants expressed preferences for AI assistance that maintained their decision-making authority:

“My AI-enabled communication aids helped me receive information during healthcare appointments, but healthcare providers sometimes directed their questions to my caregiver, rather than to me. I want the technology to facilitate communication, not replace my involvement in decisions.”

(P1: hearing impairment, Riyadh)

This perspective extends [43] the Technology Acceptance Model by revealing user preferences for AI as communication facilitator rather than decision-maker. The quote also indicates how healthcare providers’ responses to AI-mediated communication can affect user autonomy—a systemic issue beyond technology design.

#### 4.2.5. Cultural Dimensions of Autonomy

The findings suggest culturally specific dimensions of autonomy that differ from Western individualistic frameworks. In the Saudi context, personal autonomy appears to operate within family-centered healthcare decision-making structures.

Analysis indicates that autonomy in this context may mean “independence within interdependence”—the ability to contribute to family healthcare discussions rather than making unilateral decisions. This extends the work in [24] on family caregiving by examining how AI technologies can support or challenge culturally appropriate forms of autonomy.

### 4.3. Technological Barriers

This theme examines the structural and systemic barriers that limit PwDs’ ability to access and maintain AI healthcare technologies. It emerged from 31 coded segments across eight interviews, encompassing infrastructure limitations, digital literacy gaps, and financial accessibility challenges.

These findings relate to the work in [10] on digital healthcare infrastructure and extend the analysis in [28] of e-health implementation challenges in Saudi Arabia. The results contribute to discussions about healthcare AI equity [19] while examining how infrastructure barriers may disproportionately affect PwDs requiring continuous connectivity.

Geographic disparities in infrastructure access emerged as a defining factor in AI healthcare technology adoption (Table 4). Urban participants reported notably higher infrastructure reliability across all categories, with stable internet connectivity available to four of five participants compared to one of four rural participants. Technical support accessibility demonstrated the starkest disparity, with urban areas providing same-day or next-day assistance while rural participants experienced 1–2-week delays. These infrastructure gaps extended beyond individual access to include family caregiver technical literacy, where three of five urban family members demonstrated technical competence compared to one of four in rural areas.

#### 4.3.1. Infrastructure Dependencies and Geographic Inequities

Infrastructure limitations emerged as a fundamental barrier, particularly affecting participants in border regions where AI healthcare technologies require consistent connectivity:

“My wearable health monitoring device depends on constant internet connectivity to transmit vital signs to my healthcare team. In our area, frequent connection failures mean that my rehabilitation progress is not properly tracked, and personalized adjustments are not made.”

(P7: physical and chronic conditions, Northern Border)

This finding extends the infrastructure analysis in [10] by showing how AI healthcare technologies create new forms of geographic inequality. Unlike basic telehealth services that can function with intermittent connectivity, AI systems requiring real-time data transmission appear particularly vulnerable to infrastructure limitations. This suggests that the digital divide in healthcare may deepen rather than narrow with AI adoption if infrastructure disparities persist.

#### 4.3.2. Digital Literacy and Technical Support Gaps

Digital literacy emerged as a barrier distinct from general technology familiarity, with participants requiring specialized training for AI healthcare tools:

“The AI-powered navigation assistance requires significant training and customization. The technical support team visits our region rarely, and I struggle to make the necessary adjustments.”

(P2: Visual impairment, Riyadh)

This finding relates to the work in [44] on digital health literacy while revealing additional complexity specific to AI systems. The participant’s location in Riyadh—typically considered well-served—suggests that technical support gaps exist even in urban areas when specialized AI training is required. This indicates that digital literacy barriers for AI healthcare may differ qualitatively from general digital health literacy challenges.

#### 4.3.3. Financial Sustainability and Maintenance Burden

Economic barriers extended beyond initial acquisition costs to include ongoing maintenance and updates, creating sustainability challenges:

“My AI-enhanced wheelchair with autonomous navigation capabilities has transformed my independence, but the maintenance costs are substantial. When environmental sensors need recalibration or the path-planning software requires updates, I often have to delay owing to costs.”

(P4: physical disability, Al-Jouf)

This finding contributes to discussions about healthcare AI accessibility [33] by highlighting long-term cost considerations often overlooked in implementation studies. The participant’s experience suggests that AI healthcare technologies may create new forms of economic burden through ongoing maintenance requirements that differ from traditional assistive devices requiring primarily mechanical maintenance.

#### 4.3.4. Technical Complexity and User Adaptation

Participants indicated that AI systems often required technical knowledge that exceeded their existing capabilities, creating dependency on external support.

The technical complexity barrier appeared to affect participants differently based on disability type and previous technology experience. Those with physical disabilities who had extensive assistive technology experience reported easier adaptation, while participants with sensory or cognitive disabilities faced steeper learning curves. This pattern suggests that technical complexity barriers may intersect with disability type in ways not captured in general digital literacy frameworks.

#### 4.3.5. Systemic Infrastructure Dependencies

Analysis of participant experiences reveals that technological barriers often compound rather than exist independently. Infrastructure limitations affect digital literacy development (reduced online training opportunities), which in turn affects maintenance capabilities (inability to perform remote troubleshooting), which increases financial burden (requiring on-site technical visits).

This interconnection of barriers relates to the framework in [35] on digital health disparities by showing how multiple disadvantages can amplify each other in AI healthcare contexts. The systemic nature of these barriers suggests that interventions addressing single barriers may have limited effectiveness without addressing the broader infrastructure ecosystem.

### 4.4. Psychological Acceptance

This theme examines the emotional and cultural factors influencing PwDs’ willingness to adopt and maintain AI healthcare technologies. It emerged from 26 coded segments across six interviews, encompassing emotional responses to technology adoption, trust-building processes, and cultural compatibility factors.

These findings relate to [43] the Technology Acceptance Model and extend the work in [24] on cultural factors in healthcare technology adoption. The results contribute to discussions about trust in AI systems [22] while examining how cultural and religious considerations may influence acceptance patterns in non-Western contexts.

#### 4.4.1. Emotional Responses to Invasive Technologies

Participants described varied emotional responses to AI healthcare technologies, particularly those involving direct neural or physiological monitoring:

“When I first considered the brain-computer interface, I experienced profound anxiety. However, the concept of a system that reads neural signals is invasive. It took extensive counseling and meeting others using the technology before I could proceed.”

(P5: severe physical disability, Riyadh)

This finding extends [43] the Technology Acceptance Model by revealing the role of peer support in overcoming initial technology anxiety. The participant’s need for counseling and peer contact suggests that emotional barriers to AI adoption may require social rather than purely technical solutions. This indicates that psychological acceptance involves community-level processes not captured in individual-focused technology adoption models.

#### 4.4.2. Trust Development Through Transparency

Trust in AI systems emerged as closely linked to users’ understanding of system functionality and limitations:

“My AI-driven rehabilitation technology provides detailed explanations of why it’s adjusting my exercise program. Understanding the biomechanical principles behind the recommendations helps me trust and commit to the program.”

(P6: physical and neurological disabilities, Al-Jouf)

This finding relates to the work in [22] on explainable AI while revealing how transparency requirements may differ for users with specific medical needs. The participant’s emphasis on understanding “biomechanical principles” suggests that trust-building for AI healthcare may require domain-specific explanations rather than general algorithmic transparency. This extends current discussions about explainable AI by indicating that explanation quality may matter more than explanation presence.

#### 4.4.3. Cultural Integration and Family Acceptance

Cultural factors emerged as important determinants of psychological acceptance, particularly regarding family and community comfort with AI technologies:

“The voice commands for my smart home assistant include culturally appropriate terms and can be activated using traditional Arabic phrases. This cultural sensitivity made my extended family more comfortable with technology at home.”

(P8: physical and cognitive disabilities, Northern Border)

This finding contributes to discussions about culturally adaptive technology design [15] by showing how linguistic accommodation can facilitate broader family acceptance. The participant’s emphasis on “extended family” comfort indicates that psychological acceptance in collectivist cultures may require community-level rather than individual-level adaptation.

However, cultural integration appears to extend beyond linguistic accommodation to include deeper value alignment. Participants indicated preferences for AI systems that accommodate family-centered decision-making processes, with several noting the importance of technology that supports rather than bypasses traditional consultation patterns with senior family members. Additionally, references to timing considerations suggest that cultural adaptation may need to account for religious practices such as prayer schedules and fasting periods in treatment recommendations.

The study’s exploration of cultural factors remained primarily focused on language and surface-level accommodations, with participants providing limited detail about how specific Saudi cultural values—such as family hierarchy structures, collective privacy expectations, or religious compliance considerations—influence AI acceptance decisions. This represents a gap in understanding how deeper cultural integration might enhance technology adoption beyond the linguistic adaptations identified.

These findings suggest that Western frameworks focusing on individual technology acceptance may need modification for different cultural contexts, though the mechanisms of this cultural adaptation require further investigation.

#### 4.4.4. Religious Considerations in Technology Adoption

Several participants indicated that religious compatibility influenced their acceptance of AI healthcare technologies, though this emerged more through indirect references than explicit statements.

Analysis of participant responses suggests that religious considerations operate as background factors influencing acceptance decisions. While participants did not explicitly reject technologies on religious grounds, references to “traditional” approaches and family consultation patterns indicate that religious compatibility may affect adoption timing and implementation approaches.

#### 4.4.5. Trust as a Dynamic Process

Examination of participant narratives indicates that trust in AI healthcare systems develops through experience rather than initial exposure. Participants who had used systems for extended periods (6+ months) expressed different concerns than new users.

Initial concerns focused on system reliability and invasiveness, while longer-term users raised questions about dependency and system limitations. This pattern suggests that trust in AI healthcare technologies may be dynamic, requiring different support strategies at different adoption stages. This extends current trust research by indicating that sustained acceptance may involve different factors than initial adoption.

Analysis of acceptance patterns across different AI technologies reveals variation in psychological barriers:

- Brain–Computer Interfaces: highest initial anxiety but strongest eventual acceptance;
- Wearable Devices: lowest barriers but concerns about continuous monitoring;
- Smart Home Assistants: family acceptance more important than individual comfort;
- Navigation Aids: technical reliability affects trust more than privacy concerns.

The integration of themes across different AI technologies reveals patterns that inform implementation strategies. Table 5 summarizes the key themes identified in this study along with representative quotes that illustrate participants’ experiences with AI healthcare technologies.

Analysis of technology-specific challenges and recommendations (Table 6) reveals how the four themes intersect differently across AI healthcare applications.

The relationship between disability types (Table 4) and technology-specific challenges (Table 6) indicates that implementation strategies require both disability-specific and technology-specific adaptations. For instance, participants with physical disabilities using AI-enhanced wheelchairs faced maintenance and navigation challenges that differed from those experienced by participants with cognitive disabilities using smart home assistants.

**Figure 1:**
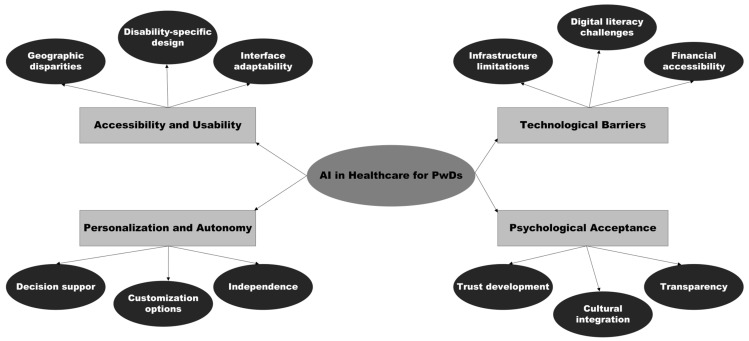
Thematic map of key findings.

The four themes operate as interconnected rather than independent factors (Figure 1). Technological barriers (Theme 3) affect psychological acceptance (Theme 4), while personalization challenges (Theme 2) influence accessibility experiences (Theme 1). This interconnection suggests that effective AI healthcare implementation requires addressing multiple themes simultaneously rather than sequential barrier removal.

These findings indicate that AI healthcare adoption for PwDs involves complex interactions between technical, cultural, and individual factors that vary across disability types, geographic locations, and technology applications. Implementation strategies may need to account for this complexity through flexible, context-sensitive approaches rather than standardized deployment models.

## 5. Discussion

This study provides insights into how people with disabilities (PwDs) experience artificial intelligence technologies in the Saudi Arabian healthcare context. Our findings from nine participants with diverse disabilities revealed important patterns in accessibility, autonomy, technological barriers, and psychological acceptance that shape AI utilization in healthcare settings.

Our findings revealed significant disparities in AI healthcare access based on geography, disability type, and socioeconomic status. The urban–rural divide was pronounced, with Northern Border participants reporting substantially lower access to AI technologies and supporting infrastructure than their Riyadh and Al-Jouf counterparts. This geographic disparity aligns with the research in [10], which identified similar patterns in digital healthcare access for PwDs in other developing healthcare contexts.

### 5.1. Conceptual Framework

**Figure 2:**
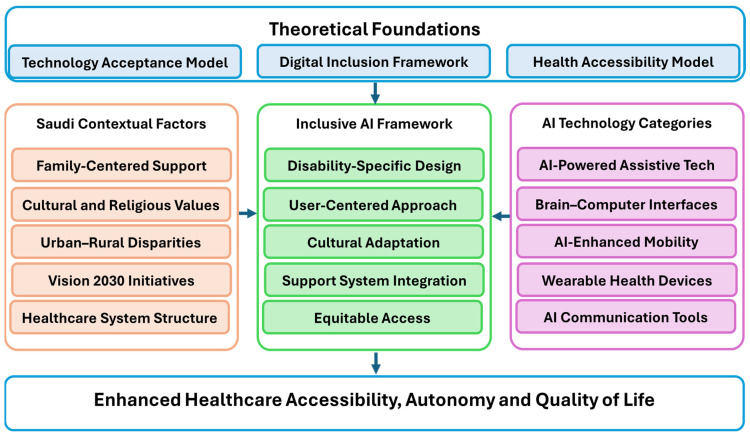
Conceptual framework for AI in disability healthcare.

According to the conceptual framework (Figure 2), this study contextualizes how AI technologies affect healthcare accessibility for PwDs in Saudi Arabia. The framework integrates theoretical foundations (Technology Acceptance Model, Digital Inclusion Framework, Health Accessibility Model), Saudi contextual factors (Vision 2030 initiatives, family-centered support, urban–rural disparities), and AI technology categories. These elements collectively influence accessibility, personalization, technological barriers, psychological acceptance, and ethics in the adoption of AI in healthcare.

### 5.2. Accessibility and Cultural Context

Despite geographic disparities, this division appears to be surprising. Some rural participants successfully accessed AI healthcare services through family support networks, suggesting that culturally adapted approaches could bridge this gap. The strong family structure in Saudi society represents a valuable resource for technology diffusion when properly engaged. This extends the work in [26] on family-centered care models by demonstrating how family networks can facilitate AI technology adoption in disability healthcare contexts.

Variations in access and usability across disability types highlight the need for disability-specific design approaches. Participants with sensory disabilities faced different challenges than those with physical, neurological, or cognitive disabilities. For example, although participants with physical disabilities benefited from AI-enhanced wheelchairs with obstacle recognition systems, those with hearing impairments required AI-enabled communication aids with different features. This diversity in needs aligns with the universal design principles advocated by [32] while emphasizing the importance of customization for unique disability-related requirements. As argued in [15], AI technologies for healthcare must be adaptable to diverse disability conditions rather than follow standardized implementation approaches.

Regional experiences within Gulf Cooperation Council countries provide relevant context for AI healthcare implementation. Ref. [28] documented similar infrastructure and cultural adaptation challenges across the region, particularly regarding Arabic language processing and family involvement requirements. Ref. [19] identified comparable barriers in middle-income countries, including connectivity limitations, digital literacy gaps, and sustainability concerns that align with findings from this study.

Cross-regional analysis suggests that technical infrastructure development alone may be insufficient for successful AI healthcare adoption among PwDs. Ref. [35] documented similar cultural and linguistic barriers in diverse populations, indicating that these challenges extend beyond the Saudi context. These regional patterns suggest that collaborative approaches addressing shared implementation barriers may enhance AI healthcare accessibility across similar cultural and economic contexts.

### 5.3. Autonomy and Agency

AI demonstrates a significant potential for enhancing autonomy among PwDs in healthcare contexts. Participants who successfully integrated AI tools, particularly brain–computer interfaces, AI-enhanced wheelchairs, and smart home assistants, reported improvements in health monitoring and independent navigation without caregiver dependency. This empowerment dimension aligns with the findings of [1], who documented similar outcomes in Western healthcare settings.

Our study reveals culturally specific dimensions of autonomy in the Saudi context. Many participants valued AI not as an alternative to family support but as a complementary resource enhancing their role within family networks. This reflects the collectivist values prevalent in Saudi society, where independence exists within interdependent family relationships rather than individual self-sufficiency. Healthcare providers and technology developers should recognize this cultural framing when implementing AI solutions in Saudi Arabia.

The tension between automated assistance and human control was significant. While valuing automation convenience, participants consistently expressed a desire to retain healthcare decision-making authority. This echoes the concerns raised by [18] regarding the balance between technological efficiency and human agencies in digital healthcare systems.

### 5.4. Technological Implementation Challenges

The identified technological barriers—infrastructure limitations, digital literacy gaps, and financial constraints—represent significant challenges to the implementation of equitable AI. Although not unique to Saudi Arabia [35], our findings revealed how these barriers manifest specifically in the Saudi context.

Infrastructure challenges were pronounced in Northern Border regions, where inconsistent Internet connectivity and limited technical support hindered the effective use of connectivity-dependent technologies, such as wearable devices and AI-enhanced wheelchairs. This aligns with the analysis in [28] of the e-health implementation challenges in Saudi Arabia. The geographic centralization of specialized healthcare services limits these limitations, making digital access particularly critical for rural residents with disabilities.

Digital literacy emerged as a significant barrier across participant groups, with many reporting insufficient training on sophisticated AI healthcare tools, particularly brain–computer interfaces and AI-powered navigation systems. This finding highlights the crucial gap in the implementation of digital health initiatives. Although Vision 2030 has accelerated technological infrastructure deployment, our results suggest that the corresponding investments in user education have not kept pace. This aligns with the critique in [29] of the implementation of mHealth in Saudi Arabia.

Financial accessibility is another significant barrier, particularly for advanced assistive devices not covered by insurance. The high costs associated with AI-enhanced wheelchair maintenance and brain–computer interface calibration are frequently cited concerns. Current healthcare financing mechanisms may not adequately account for the long-term benefits of AI-enhanced assistive technologies. Policy reforms that recognize these technologies as essential medical interventions can significantly enhance access.

### 5.5. Psychological and Cultural Acceptance

Cultural factors significantly influence AI acceptance in the healthcare context. Integrating cultural and religious considerations, such as culturally appropriate voice commands by smart home assistants, enhanced the acceptance of AI healthcare tools among participants and families. This underscores the importance of culturally sensitive design approaches with respect to local values, as advocated by [19] in their study on digital health sustainability in diverse contexts.

The psychological journey of trusting AI in healthcare has emerged as a complex process, influenced by transparency, previous technology exposure, and perceived alignment with personal values. Many participants described their initial skepticism toward technologies such as brain–computer interfaces, followed by gradual acceptance as they gained experience. This aligns with the technology acceptance models described by [43] but highlights specific concerns relevant to PwDs, particularly regarding invasive technologies.

Trust in an AI system is strongly linked to its transparency. The participants expressed greater comfort with the AI tools, which explained their reasoning and limitations. This resonates with the ethical principles of explainable AI advocated by [33] while emphasizing the importance of transparency for vulnerable populations who may have experienced healthcare marginalization.

### 5.6. Limitations and Risks of AI Healthcare Implementation

AI healthcare implementation presents risks that require consideration alongside potential benefits. Reduced human interaction may conflict with Saudi cultural values emphasizing personal relationships in medical care [40]. Participants reported concerns about decreased face-to-face contact with healthcare providers when using AI-mediated systems, aligning with findings from [18] regarding healthcare professionals’ concerns about diminished patient relationships.

Economic implications include potential displacement of healthcare support roles traditionally filled by family caregivers. Ref. [24] demonstrated that family caregivers provide essential cultural interpretation and emotional support functions that extend beyond basic assistance. AI automation of these roles may undermine established caregiving structures without adequate replacement mechanisms.

Technical dependency risks emerged through participant experiences with system failures and maintenance challenges. Ref. [19] identified similar sustainability concerns in low- and middle-income countries, where technical infrastructure limitations can transform digital health solutions into access barriers. Over-reliance on AI systems may reduce individual self-advocacy capabilities, particularly problematic in rural areas with limited technical support [28].

Privacy considerations specific to collectivist cultures warrant attention. Current AI systems designed for individual users may inadequately accommodate family-centered decision-making processes prevalent in Saudi healthcare contexts [26].

### 5.7. Ethical Considerations for AI Healthcare Implementation

The findings reveal ethical challenges in AI healthcare implementation for PwDs that differ from standard AI ethics frameworks. These challenges suggest that ethical AI implementation may require context-specific approaches alongside universal principles.

Traditional AI ethics frameworks emphasize individual autonomy and privacy, yet participants indicated preferences for family-involved healthcare decisions. This creates tension between individual consent principles and Saudi cultural values emphasizing collective family decision making. The disconnect raises questions about consent requirements for AI healthcare data collection and how privacy should be defined when healthcare decisions involve family consultation.

Participants with different disabilities experienced varying levels of AI system effectiveness, suggesting potential algorithmic bias. Current fairness metrics may not capture disability-specific performance disparities, particularly when AI systems perform adequately for common disability presentations but show reduced effectiveness for complex or multiple disabilities. This pattern indicates that algorithmic bias in AI healthcare may require disability-specific fairness assessments.

Geographic and economic barriers identified in the study raise justice concerns about equitable access to AI healthcare benefits. The differential availability of infrastructure and technical support across regions creates disparities that may increase rather than reduce health inequities for PwDs in marginalized communities. These access barriers intersect with disability status to create compounded disadvantages.

Participants’ references to cultural and religious considerations indicate that ethical AI healthcare implementation may require accommodation of religious practices and cultural values. Current AI ethics frameworks provide limited guidance for integrating such accommodations while maintaining system effectiveness and fairness.

These challenges suggest that AI healthcare implementation for PwDs in diverse cultural contexts may require frameworks that address both universal ethical principles and context-specific cultural values. The findings indicate that ethical AI healthcare development may benefit from inclusive design processes involving local communities and disability advocates.

### 5.8. Implications for Policy and Practice

This study has significant implications for Saudi Arabia’s Vision 2030 healthcare transformation. While participants appreciated progress in digital healthcare infrastructure, they identified critical gaps that must be addressed to realize AI’s potential for PwDs.

The integration of AI into disability healthcare aligns with the key Vision 2030 objectives, which include enhancing healthcare quality and accessibility. However, achieving these objectives requires targeted strategies to address the barriers identified in this study.

Based on our analysis of specific AI technologies and their challenges, we recommend:

- Developing comprehensive accessibility standards for national health applications and AI systems;
- Establishing subsidized access programs for AI-enhanced assistive technologies, particularly in rural areas;
- Creating specialized digital literacy programs tailored to different disability types;
- Incentivizing culturally appropriate design in healthcare AI development;
- Integrating disability perspectives into healthcare AI implementation.

## 6. Limitations

This study’s sample of nine participants limits generalizability to the broader PwDs population in Saudi Arabia. While purposive sampling enabled detailed exploration of individual experiences, the sample size restricts representation across disability types, geographic regions, and socioeconomic backgrounds. Participants were drawn from three regions (Riyadh, Al-Jouf, Northern Border), providing initial insights but potentially missing perspectives from other Saudi regions with different infrastructure and cultural contexts.

The qualitative approach prioritized depth over breadth, yielding detailed narratives about specific AI healthcare adoption challenges and facilitators. However, findings should be interpreted as exploratory rather than definitive, particularly regarding technology-specific recommendations and cultural adaptation strategies. Additionally, the cultural analysis focused primarily on linguistic accommodation rather than deeper integration of cultural values such as family-centered decision-making processes, collective privacy expectations, and religious practice accommodation.

## 7. Conclusions

This qualitative study examined AI healthcare experiences among nine PwDs across three Saudi regions. The findings revealed four themes: accessibility challenges, personalization needs, technological barriers, and psychological acceptance factors. Cultural values, particularly family-centered decision making, influenced technology adoption patterns.

Key contributions include the documentation of cultural factors affecting AI acceptance in Saudi healthcare contexts and identification of rural implementation challenges requiring targeted interventions. Results indicate that technology deployment alone may be insufficient without corresponding infrastructure, training, and cultural adaptation measures.

Implementation recommendations include developing accessibility standards for national health applications, establishing subsidized access programs for rural areas, creating disability-specific digital literacy programs, and integrating cultural considerations into AI healthcare design.

### Future Research Directions

Several research gaps warrant investigation to advance AI healthcare implementation for PwDs. Larger studies across additional Saudi regions, encompassing diverse disability types, age groups, and socioeconomic backgrounds, would enhance understanding of adoption patterns and improve the transferability of findings. Longitudinal research should examine sustained AI adoption and long-term outcomes across different contexts.

Cost-effectiveness analyses of AI healthcare interventions, including maintenance costs and infrastructure requirements, would inform policy decisions. Comparative studies across Gulf Cooperation Council countries could provide regional implementation insights and identify shared strategies for addressing common barriers.

Future work should explore cultural integration beyond linguistic accommodation, investigating how AI systems can accommodate family-centered healthcare decisions and religious considerations. Mixed-methods studies combining quantitative adoption metrics with qualitative user experiences would provide robust evidence for implementation strategies. Research examining optimal training methodologies for different disability types and digital literacy levels could inform targeted intervention development.

Ethical studies should address consent and privacy considerations in family-centered healthcare decision-making contexts. These research directions would support AI healthcare implementation within Saudi Arabia’s Vision 2030 transformation framework.

## Figures and Tables

**Table 1:** Demographic profile of participants.

ID	Gender	Age	Region	Type of Disability	AI Technology	Digital Literacy
P1	F	29	Riyadh	HI	AI-ECA	High
P2	M	45	Riyadh	VI	AI-PNA	Moderate
P3	F	32	Riyadh	PD	AI-PAT	High
P4	M	38	Al-Jouf	PD	AI-EWM	High
P5	F	27	Riyadh	SPD	BCI	High
P6	M	41	Al-Jouf	PND	AI-DRT	Moderate
P7	F	34	Northern Border	PCC	WD	Moderate
P8	M	52	Northern Border	PCD	SHA	Low
P9	F	44	Northern Border	SI	AI-ECA	Low

Note: F = female; M = male; HI = hearing impairment; VI = visual impairment; PD = physical disability; SPD = severe physical disability; PND = physical and neurological disability; PCC = physical and chronic condition; PCD = physical and cognitive disability; SI = speech impairment; AI-ECA = AI-enabled communication aid; AI-PNA = AI-powered navigation assistance; AI-PAT = AI-powered assistive technology; AI-EWM, AI-enhanced wheelchair mobility; BCI, brain–computer interface; AI-DRT, advanced rehabilitation technology; WD, wearable device; SHA, smart home assistant.

**Table 2:** Interview items.

Question Category	Questions	Purpose	Key References
Digital Healthcare Access	1. How do you currently access healthcare services using digital technology?	To understand current utilization patterns	[20]
	2. What types of AI healthcare tools do you use most frequently?	To identify technology preferences	[16]
Technology Usage	1. How comfortable are you with using AI healthcare applications?	To assess digital literacy	[32]
	2. What difficulties do you experience when using these technologies?	To identify barriers	[35]
Healthcare Provider Interaction	1. How do you communicate with healthcare providers using AI technologies?	To understand provider-patient interaction	[17]
	2. What has been your experience with telehealth appointments?	To assess telehealth experiences	[36]
Cultural and Contextual Factors	1. How do your family members view your use of AI healthcare tools?	To explore cultural perspectives	[24]
	2. How do religious or cultural considerations affect your use of AI tools?	To identify cultural integration needs	[19]

Note: Questions were presented in Arabic during interviews. AI = artificial intelligence.

**Table 3:** Types of disabilities and AI technologies utilized.

Disability Type	*n*	AI Technologies	Key Applications
PD	2	AI-PAT, AI-EWM	Smart wheelchairs with obstacle recognition, autonomous wheelchairs with adaptive controls, path planning, and navigation assistance
SPD	1	BCI	Direct neural control for operating mobility and assistive devices
VI	1	AI-PNA	Object recognition, environmental scanning, audio descriptions of surroundings
PCC	1	WD	Fitness tracking tailored to abilities, continuous health monitoring, personalized rehabilitation
PND	1	AI-DRT	Personalized exercise programs, real-time feedback on rehabilitation progress
PCD	1	SHA	Voice-activated systems for controlling appliances, setting reminders for appointments
HI	1	AI-ECA	Speech-to-text conversion, audio enhancement, visual alerts for auditory information
SI	1	AI-ECA	Specialized speech recognition, text-to-speech conversion, alternative communication interfaces

Note: PD = physical disability; SPD = severe physical disability; VI = visual impairment; PCC = physical and chronic conditions; PND = physical and neurological disability; PCD = physical and cognitive Disability; HI = hearing impairment; SI = speech impairment; AI-PAT = AI-powered assistive technology; AI-EWM = AI-enhanced wheelchair mobility; BCI = brain–computer interface; AI-PNA = AI-powered navigation assistance; WD = wearable device; AI-DRT = AI-driven rehabilitation technology; SHA = smart home assistant; AI-ECA = AI-enabled communication aid.

**Table 4:** Infrastructure challenges by geographic location.

Challenge Category	Urban Areas (n = 5)	Rural Areas (n = 4)
Internet connectivity reliability	4/5 reported stable	1/4 reported stable
Technical support availability	Same-day or next-day	1–2 weeks wait time
Device maintenance access	Local repair centers	Regional travel required
Digital literacy support	Community centers available	Limited or no programs
Healthcare system integration	Full platform access	Intermittent connectivity
Family caregiver technical knowledge	3/5 family members tech-literate	1/4 family members tech-literate

Note: data based on participant self-reports during interviews.

**Table 5:** Summary of key themes and participant quotes.

Theme	Key Subthemes	Representative Quote
AU	Geographic disparities, disability-specific design, interface adaptability	“My smart wheelchair with AI obstacle recognition has transformed my hospital visits. The integration with the hospital’s navigation system guides me directly to my appointments.” (P3)
PA	Customization options, independence, decision support	“My brain-computer interface system has given me unprecedented independence. I can control my medical devices, communicate with healthcare providers, and access my health records using just my thoughts.” (P5)
TB	Infrastructure limitations, digital literacy, financial accessibility	“My wearable health monitoring device depends on constant internet connectivity to transmit vital signs to my healthcare team. In our area, frequent connection failures mean my rehabilitation progress isn’t properly tracked, and personalized adjustments aren’t made.” (P7)
PSA	Trust development, transparency, cultural integration	“The voice commands for my smart home assistant include culturally appropriate terms and can be activated using traditional Arabic phrases. This cultural sensitivity made my extended family more comfortable with the technology in our home.” (P8)

Note: AU = accessibility and usability; PA = personalization and autonomy; TB = technological barrier; PSA = psychological acceptance.

**Table 6:** AI technology-specific challenges and recommendations matrix.

Technology	Challenges	Recommended Solutions
AI-PAT	Insufficient training data for diverse disability presentations	Develop inclusive datasets representing both urban and rural users
	Environmental limitations affecting sensing capabilities	Design adaptable sensing systems for both urban and rural environments
	Limited maintenance support in rural areas	Create tiered support systems with enhanced remote options for rural users
BCI	Data privacy concerns in collective family settings	Develop specialized encryption with cultural privacy considerations
	Limited specialist support outside major urban centers	Establish hub-and-spoke telemedicine counseling programs
	Religious and cultural concerns about mind-technology integration	Create culturally sensitive implementation guidelines with religious scholars’ input
AI-EWM	Navigation system variations between urban and rural environments	Develop adaptive mapping capabilities with region-specific terrain data
	Maintenance cost disparities between regions	Implement geographically adjusted subsidized maintenance programs
	Technical support accessibility differences	Train local technical support personnel with region-specific knowledge
WD	Connectivity disparities between urban and rural regions	Design devices with connectivity-adaptive functionality
	Device adaptability across diverse user contexts	Implement modular designs with customizable components
	Digital literacy variations affecting data interpretation	Develop tiered visualization approaches based on user literacy levels
SHA	Urban–rural emergency service integration differences	Create location-aware healthcare vocabulary and command sets
	Reliability variations in alert systems across regions	Establish region-specific notification standards and protocols
	Family privacy expectations in collective households	Develop culturally appropriate privacy settings with regional customization

Note: AI-PAT = AI-powered assistive technology; BCI = brain–computer interface; AI-EWM = AI-enhanced wheelchair mobility; WD = wearable device; SHA = smart home assistant.

## Data Availability

The datasets used and/or analyzed during the current study are available from the corresponding author upon reasonable request.
